# MTRX1011A, a humanized anti-CD4 monoclonal antibody, in the treatment of patients with rheumatoid arthritis: a Phase I randomized, double-blind, placebo-controlled study incorporating pharmacodynamic biomarker assessments

**DOI:** 10.1186/ar3502

**Published:** 2011-10-26

**Authors:** Heleen Scheerens, Zheng Su, Bryan Irving, Michael J Townsend, Yanan Zheng, Eric Stefanich, Vishala Chindalore, Clifton O Bingham, John C Davis

**Affiliations:** 1 Genentech Research and Early Development, 1 DNA Way, South San Francisco, CA 94080, USA; 2 Pinnacle Research Center Anniston Medical Clinic PC, 1010 Christine Avenue, Anniston, AL 36207, USA; 3 Allergy and Clinical Immunology, Johns Hopkins University, 5200 Eastern Avenue, MFL Center 404, Baltimore, MD 21224, USA

**Keywords:** rheumatoid arthritis, pharmacodynamics, phase I, antibody

## Abstract

**Introduction:**

The purpose of this study was to evaluate the safety, tolerability, pharmacokinetics (PK) and pharmacodynamics (PD) of the humanized anti-CD4 monoclonal antibody MTRX1011A in a randomized, double-blind placebo-controlled Phase 1 study in patients with rheumatoid arthritis (RA).

**Methods:**

In the single ascending dose (SAD) portion of the study, patients received single doses of a placebo or MTRX1011A at 0.3, 1.0, 3.5 and 7.0 mg/kg intravenously (IV) or 1.0 and 3.5 mg/kg subcutaneously (SC), followed by five weeks of evaluation. In the multi-dose (MD) portion of the study, placebo or MTRX1011A was administered weekly for eight doses at 1.5 or 3.5 mg/kg SC, or 5 mg/kg IV, followed by eight weeks of evaluation.

**Results:**

MTRX1011A was well tolerated in the SAD phase up to 7 mg/kg IV and in the MD phase up to 1.5 mg/kg SC. At weekly doses of 3.5 mg/kg SC and 5 mg/kg IV, a moderate pruritic papular rash was observed in some MTRX1011A-treated patients, which was considered a dose-limiting toxicity for this clinical indication. No serious adverse events occurred in any cohort. Reduction in disease activity was modest. PD assessments demonstrated that MTRX1011A induced a dose-dependent down-modulation of CD4 expression on peripheral blood CD4 T cells, CD4 receptor occupancy, increases in serum sCD4-MTRX1011A complexes and up-regulation of CD69 on T cells, but was non-depleting.

**Conclusions:**

The maximum tolerated dose of MTRX1011A was 1.5 mg/kg SC administered weekly. At this dose MTRX1011A did not achieve maximum PD activity expected to be required for reduction in disease activity.

## Introduction

Although the etiology and pathogenesis of rheumatoid arthritis (RA) remain to be fully elucidated, the disease is characterized in part by a cell-mediated immune response. Many novel therapeutics have attempted to target cell-mediated pathways, including those targeting CD4 T cells. The first line of treatment typically involves the use of disease-modifying anti-rheumatic drugs (DMARDs). Biologics may be subsequently added to the treatment repertoire in inadequate responders. Despite these treatments available for RA, a significant number of patients are unresponsive or intolerant to current therapies, and a significant need remains for novel effective treatments for RA [1,2].

A critical role of CD4 T cells in the pathogenesis of RA has been described by multiple groups. Increased numbers of CD4 T cells are detected in inflamed RA synovium, elevated levels of activated T cells in the peripheral blood of RA patients are observed, and disease susceptibility is associated with certain major histocompatibility complex class II (MHCII) alleles [3-6]. Preclinical studies with anti-CD4 therapeutics have provided further evidence for the critical role of CD4 T cells in the pathogenesis of disease [7]. Abatacept is an approved therapeutic for patients with RA that reduces disease activity by blocking the CD80/CD86:CD28 co-stimulation signal of CD4 T cells [8]. MTRX1011A is a humanized IgG1 anti-CD4 monoclonal antibody (MAb) derived from a previously described TRX1 antibody [9]. It binds with high affinity to human CD4 T cells with an equilibrium dissociation constant (K_D_) less than 1 nM. MTRX1011A down-modulates cell surface expression of CD4 and inhibits the function of residual surface CD4 by blocking its interaction with MHC II. An amino acid substitution of N297A was introduced to impair binding to Fcγ receptors and consequently prevent Fc-mediated effector function [10,11], rendering the antibody non-depleting *in vivo *[12,13]. In MTRX1011A, an additional single amino acid substitution was made in the Fc region of the antibody (N434H) to improve its binding to the neonatal Fc receptor (FcRn) [14]. This improved binding to FcRn was expected to enhance antibody recycling from the endosome back to the circulation and protect it from degradation in the lysosome, therefore decreasing MTRX1011A *in vivo *clearance [14].

Several prior therapeutics targeting the CD4 molecule have been reported. Studies examining the anti-CD4 antibodies keliximab, clenoliximab, and 412W94, resulted in varying levels of clinical response, suggesting that CD4 may represent a valid target for the treatment of RA [15-17]. Differences in RA patient populations studied and dosing regimens employed might account for the different clinical outcomes observed; in addition keliximab, 412W94, and cM-T412, a fourth anti-CD4 antibody evaluated in RA patients, depleted peripheral CD4 T cells [18,19]. A dose-limiting rash was observed in several studies with both depleting and non-depleting anti-CD4 antibodies [15,16,20]; however, detailed descriptions and evaluations of these rashes were limited. The efficacy of non-depleting anti-CD4 antibodies is thought to be mediated by down-modulation of the CD4 receptor on T cells through internalization of the antibody-receptor complex and subsequent blocking of the interaction of the remaining CD4 co-receptor with MHCII on antigen presenting cells, resulting in reduced T cell activation. Pharmacodynamic (PD) evaluations with cM-T412 in patients with RA suggested that sustained maximum CD4 occupancy on peripheral blood T cells was required to induce CD4 occupancy on T cells in the synovium [21]. Studies by Mason *et al*. suggested that sustained CD4 down-modulation and CD4 receptor occupancy are required for non-depleting anti-CD4 antibodies to be effective [15]. Furthermore, studies in collagen-induced arthritis mouse models demonstrated that CD4 T cell depletion is not required for efficacy [7], and this was subsequently confirmed in clinical studies with clenoliximab. Taken together, these studies suggest that clinical efficacy from anti-CD4 antibodies could be achieved by maintaining maximum CD4 occupancy and down-modulation throughout the dosing period.

We report the results of a phase I study in which the safety, tolerability, pharmacokinetics (PK) and PD of MTRX1011A were evaluated in RA patients. The aim of this study was to identify a dose of MTRX1011A that was safe, well tolerated and maintained maximum CD4 occupancy and down-modulation that was not associated with rash. Extensive exploratory biomarkers were incorporated to investigate the mechanism of action of MTRX1011A.

## Materials and methods

### Patients

Sixty-six RA patients were enrolled at 16 study sites in the US. All sites were Institutional Review Board-approved, and all patients provided voluntary written informed consent (Registered at ClinicalTrials.gov; NCT00718588). Eligible patients were between the ages of 18 and 80 years and fulfilled the American College of Rheumatology (ACR) 1987 revised criteria for RA. Enrollment criteria required patients to have CD4 T cell counts above the lower limit of normal, to be on a stable regimen of anti-rheumatic therapy (four to eight weeks for DMARDS; two weeks for nonsteroidal anti-inflammatory drugs (NSAIDs), four weeks for steroids), and to have an appropriate washout period after discontinuation of any previous biologic treatments. Additionally, patients enrolled in the multiple dose (MD) phase of the study were required to have at least three swollen and tender joints and had shown an inadequate response or intolerance to at least one biological agent. Significant exclusion criteria were recurrent or chronic infection, and any significant medical condition that would predispose to an increased risk by participation in a clinical trial, and the presence of other autoimmune disease with the exception of secondary Sjögren's syndrome.

### Study design

In the single ascending dose (SAD) phase, 30 patients were treated in six cohorts of 5 patients each (1 randomized to placebo and 4 randomized to MTRX1011A), with four IV doses (0.3, 1.0, 3.5 and 7.0 mg/kg) and two SC dose levels (1.0 and 3.5 mg/kg) of MTRX1011A. Dose escalation occurred after safety was established for at least 14 days from the previous cohort. Initiation of the MD phase of the study was dependent upon acceptable safety data from the SAD phase up to 14 days of all patients in the high dose SC and IV cohorts. Thirty-six patients were randomized into the MD phase of the study and received MTRX1011A or placebo weekly for eight doses. Two SC cohorts consisted of 12 patients receiving MTRX1011A (at 1.5 mg/kg and 3.5 mg/kg) and 3 receiving placebo weekly. One IV cohort consisted of five patients receiving MTRX1011A (at 5.0 mg/kg) and one receiving placebo weekly. Patients were followed for five or eight weeks following dose administration in the SAD and MD phase respectively.

### Clinical evaluations

The primary outcome measures of the study were the safety and tolerability. Exploratory outcome measures in the multi-dose phase of the study included the proportion of patients who achieved ACR20/50/70, and several patient-reported outcome measures. Dermatologic consultation, photography and skin biopsy were encouraged if patients experienced rash.

### Pharmacokinetics

Serum samples were collected to measure the concentration of total MTRX1011A and to detect the presence of potential anti-therapeutic antibodies by ELISA as previously described [14].

### Pharmacodynamics

Whole blood samples were collected for flow cytometric analysis of lymphocyte subsets. First, a standard T, B, NK flow cytometry panel (BD Biosciences (San Jose, CA, USA) was used for cell quantification. Due to interference of MTRX1011A with the anti-CD4 antibody in the flow cytometry panel, CD4 T cells were identified as CD3+CD8- T cells and in an additional exploratory panel using an anti-CD4 antibody (clone MT-441) in which binding was not affected by MTRX1011A. Second, to evaluate internalization of CD4 by MTRX1011A, expression levels of CD4 were determined using the non-competing anti-CD4 antibody (clone MT-441). Receptor occupancy of CD4 was determined by staining with fluorescently labeled MTRX1011A. CD4 expression and occupancy data were expressed as change from pre-dose values (% baseline) for placebo and MTRX1011A groups. Lastly, T cell subsets were classified into memory and naïve subsets using CD45RA antibody, and the expression of the activation markers CD25 and CD69 was characterized. B cells subsets were enumerated by naïve and memory subpopulations using CD27, CD38, and IgD surface markers.

Serum levels of total soluble CD4 and soluble CD25 were determined by ELISA. Serum levels of pro-inflammatory cytokines TNF-α, IL-6, GM-CSF, IFNγ, IL-10, IL12p70, IL-1β, IL2, and IL-8 were measured using multiplex electrochemiluminescence assays on the Meso Scale Discovery SECTOR Imager 6000 platform (Gaithersburg, MD, USA).

### Statistical analysis

All data analyses were conducted using descriptive statistics, which were based on the intent-to-treat principle. Results were summarized separately for the placebo group (pooled across all dosage levels) and for each of the nine MTRX1011A cohorts. The sample size for the SAD phase was chosen to detect major intolerability signals. The sample size in the MD phase (the 1.5 and 3.5 mg/kg SC cohorts) was chosen to better understand the PK and PD properties of MTRX1011A and to explore possible clinical benefit of MTRX1011A.

## Results

### Patient baseline demographics, characteristics, and disposition

**Figure 1:**
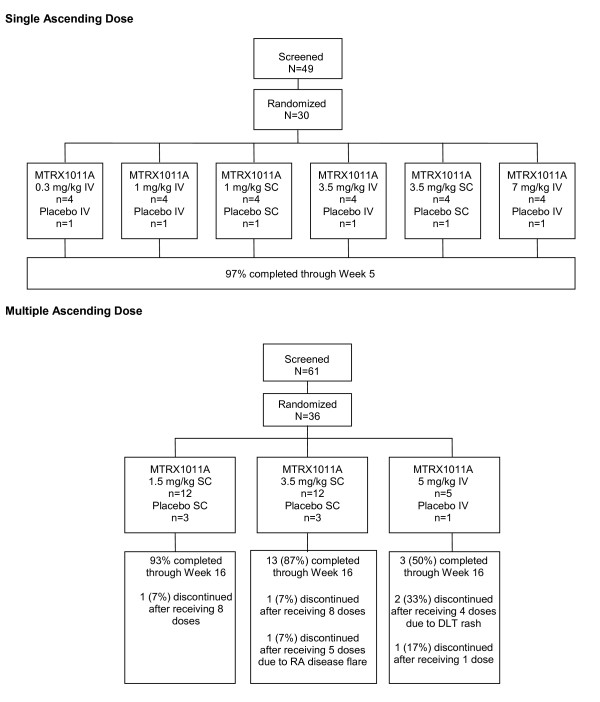
**Patient disposition**. Single ascending dose and multiple dose stages.

Patient disposition is reflected in Figure 1. A total of 66 patients were randomized, and their baseline characteristics are presented in Table 1. There were no significant differences in baseline characteristics between any of the treatment groups. The majority of patients were Caucasian females, mean age for all randomized patients was 54 years, and was balanced between groups. The mean disease duration ranged from 3.8 to 8.1 years. Patients in the MD phase had higher C-reactive protein) CRP levels, erythrocyte sedimentation rate (ESR) and tender joint count (TJC) than patients in the SAD phase, reflective of the differences in enrollment criteria for the SAD and MD phases. Baseline Disease Activity 28 joints-C-reactive protein scores (DAS28(4)-CRP) scores ranged from 4.8 to 5.9. Patients in the MD phase had slightly lower incidence of RF positivity than patients in the SAD phase, despite long standing disease and 100% anti-cyclic citrullinated peptide (CCP) positivity.

**Table 1:** Demographics and baseline characteristics of the patients

	Single dose phase		Multi dose phase	
Characteristics	Placebo	MTRX1011A	Placebo	MTRX1011A
Gender (Female:Male)	4:2	15:9	6:1	24:5
Age, years Mean (range)	51 (41 to 64)	56 (27 to 77)	57 (43 to 57)	53 (25 to 76)
Race (White:Black)	5:1	23:1	7:0	24:5
Disease Duration (median years)	3.8	5.7	5.0	8.1
RF positive, %	66.7	58.3	42.9	34.5
CCP positive, %	100	100	100	100
RF and anti-CCP positive, %	66.7	58.3	42.9	34.5
Concomitant				
Meds:				
% on MTX	67	75	57	66
% on	0	4	14	0
leflunomide % on steroids	33	50	43	48
CRP, mg/dL Mean (range)	0.53 (0.50 to 0.93)	0.65 (0.04 to 2.89)	1.68 (003 to 3.99)	1.11 (0.02 to 8.21)
ESR, mm/hour Mean (range)	26 (0 to 42)	25 (4 to 49)	47 (15 to 120)	40 (0 to 110)
SJC Mean (range)	17 (10 to 33)	17 (0 to 63)	7 (0 to 16)	18 (2 to 59)
TJC Mean (range)	25 (6 to 44)	31 (1 to 68)	36 (10 to 53)	43 (58 to 68)
Baseline DAS-CRP	4.8 (3.85 to 5.68)	5.15 (2.66 to 7.17)	5.43 (4.28 to 6.62)	5.93 (3.74 to 8)

anti-CCP, anti-cyclic citrullinated peptide antibodies; CRP, C-reactive protein; DAS, disease activity score; ESR, erythrocyte sedimentation rate; MTX, methotrexate; RF, rheumatoid factor; SJC, swollen joint count; TJC, tender joint count; TNF, tumor necrosis factor.

RF positive was defined as RF ≥20 IU/ml; anti-CCP positive was defined as anti-CCP ≥5 U/ml.

Five patients withdrew early from the study, four of those were based on decisions by the patient, and one patient was dosed SC instead of IV in the last cohort and was withdrawn from the study due to increased disease activity judged by the site investigator several days later. This patient was replaced according to protocol specified guidance. However, dosing was later discontinued in the 5 mg/kg IV weekly cohort per protocol rules secondary to dose-limiting toxicities of rash.

### Safety

Adverse events (AEs) reported in ≥ 2 patients are shown in Table 2. The overall incidence of AEs was higher in patients treated with MTRX1011A than in those treated with placebo.

**Table 2:** Adverse events in ≥2 patients

	SAD Stage		MD Stage				
No. (%) Patients	Placebo (*n *= 6)	All Active (*n *= 24)	Placebo (*n *= 7)	1.5 mg/kg (SC) (*n *= 12)	3.5 mg/kg (SC) (*n *= 12)	5.0 mg/kg (IV)(*n *= 5)	All Active(*n *= 29)
**Adverse Events**							
Any event	1 (17)	6 (25)	0 (0)	1 (8)	3 (25)	1 (20)	5 (17)
Nausea	0 (0)	2 (8)	0 (0)	0 (0)	1 (8)	0 (0)	1 (3)
Flank pain	0 (0)	2 (8)	0 (0)	0 (0)	0 (0)	0 (0)	0 (0)
Fatigue	0 (0)	0 (0)	0 (0)	1 (8)	1 (8)	0 (0)	2 (7)
Pruritis	0 (0)	0 (0)	0 (0)	0 (0)	1 (8)	1 (20)	2 (7)
Rash	0 (0)	0 (0)	0 (0)	0 (0)	1 (8)	1 (20)	2 (7)
**Serious Adverse Events**							
Any event	0 (0)	0 (0)	0 (0)	0 (0)	0 (0)	0 (0)	0 (0)

IV, intravenous; MD, multiple dose; SAD, single ascending dose; SC, subcutaneous.

*Adverse events determined by investigators to be related to MTRX1011A by preferred term in ≥ 2 patients.

SAD Phase: Six patients (25.0%) receiving MTRX1011A experienced at least one drug-related AE compared with one (16.7%) patient who received placebo. One patient in the SAD phase who received MTRX1011A experienced pruritis one day after dosing that resolved spontaneously.

MD Phase: Five patients (17.2%) receiving MTRX1011A experienced at least one drug-related AE compared with no patients receiving placebo. Gastrointestinal disorders were reported only in MTRX1011A-treated patients (27.6%) and were mild. One MTRX1011A-treated patient in the MD phase 3.5 mg/kg SC cohort experienced oral candidiasis (considered a dose limiting toxicity) and a moderate papular pruritic rash both during and after dosing.

Because an increased incidence of rash was observed in previous anti-CD4 clinical studies, special consideration was given to cutaneous events in the study. In the MD phase, 10 (34.5%) MTRX1011A-treated patients versus no placebo patients reported cutaneous disorders overall. There were no cases of drug-related rash in the 1.5 mg/kg SC cohort; however, one patient in this cohort experienced pruritis. In the majority of the cases in the higher dose cohorts, the rash was observed after patients received three or four doses (3 out of 12 active treatment subjects in the 3.5 mg/kg SC cohort and 2 out of 4 active treatment subjects in the 5.0 mg/kg IV cohort). In all cases, the rash resolved when MTRX1011A was discontinued or the rash was treated with topical or oral agents (for example, antihistamines and/or corticosteroids). The rash was usually preceded by the onset of pruritis at least several hours to days before the visible rash. In two patients, the rash recurred with re-exposure to the drug, in one after receiving the last dose and in the other after each consecutive dose of MTRX1011A. The rash was widely distributed throughout the body -- on the abdomen, back, and extremities. In most cases the rash was erythematosus, raised, papular and intensely pruritic (resulting in excoriations in several patients). In two of the patients, a dermatologic consultation and skin biopsy was obtained. One biopsy from a patient in the MD 3.5 mg/kg SC cohort revealed a superficial perivascular infiltrate of lymphocytes, histiocytes and a few eosinophils in the dermis. The epidermis demonstrated spongiosis with overlying compact hyperkeratosis and focal parakeratosis. The other biopsy was collected from a patient in the MD 5 mg/kg IV cohort, and revealed palisaded neutrophilic and granulomatous dermatitis with necrobiosis of collagen fibers in the upper dermis.

### Laboratory parameters

**Figure 2:**
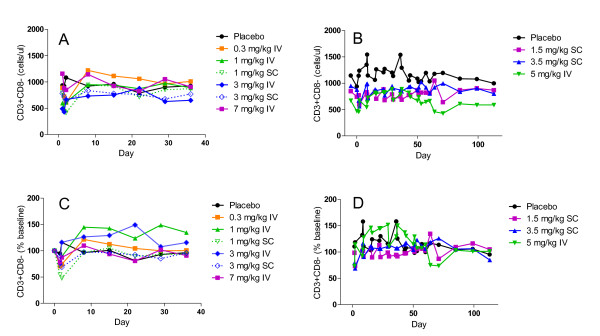
**Peripheral blood T cell levels**. Blood CD4 T cells in patients after a single dose (A and C) or 8 weekly doses (B and D) of MTRX1011A in doses ranging from 0.3 to 7.0 mg/kg. Mean CD4 T cells are expressed as cells/μl (A and B) or as % baseline (C and D).

No pattern of clinically significant changes was observed for the hematology, chemistry, and urinalysis parameters in any cohort. Additionally, no sustained pattern of CD4^+ ^T-cell depletion was observed in any cohort. The mean peripheral blood CD4 T cell counts are presented in Figure 2. In some patients a transitory CD4 T cell reduction was observed immediately following administration of MTRX1011A. CD4 T cell counts < 250 cells/μl were detected in 10 patients 4 hr after dosing and in 3 patients 24 hr after dose administration. No apparent safety-related significance was attributed to this transient reduction in CD4 T cell counts, and prior to the second dose administration CD4 T cell counts were above 250 cells/μl in all patients.

### Immunogenicity

Anti-therapeutic antibodies were detected post dose in three MTRX1011A treated patients. The systemic exposure to MTRX1011A in these three patients was similar to the systemic exposure to MTRX1011A in the other patients in the respective cohorts, indicating that the presence of anti-MTRX1011A antibodies did not appear to impact the systemic exposure to MTRX1011A.

### Pharmacokinetics

MTRX1011A exhibited non-linear PK properties in the dose ranges tested, as was expected for an antibody with high target mediated clearance (CL). The mean total CL ranged from 79 to 8.1 ml/kg/day for the 0.3 to 7.0 mg/kg single dose IV cohorts, respectively, and 6.2 ml/kg/day for the 5 mg/kg multi-dose IV cohort. Absolute SC bioavailability was estimated to be approximately 52%. Modest accumulation was observed upon multiple weekly dosing at 1.5 to 5 mg/kg SC or IV [14].

### Pharmacodynamics

#### CD4 expression and occupancy

**Figure 3:**
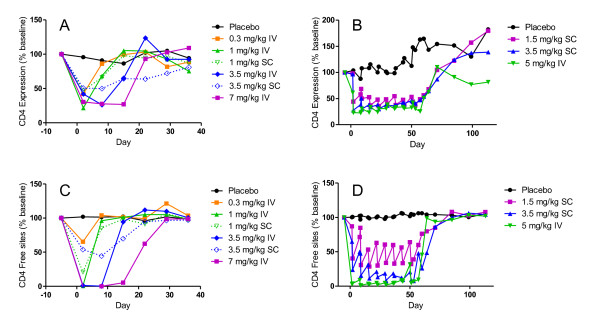
**Peripheral blood CD4 expression and CD4 occupancy**. Mean changes in peripheral blood CD4 expression and CD4 occupancy on memory (CD45RA-) CD4 T cells in patients receiving placebo or MTRX1011A in the single dose (A and C) and multiple dose (B and D) phase of the study when administered intravenously (IV) or subcutaneously (SC) at doses ranging from 0.3 to 7 mg/kg. A and B, MTRX1011A treatment induced a dose dependent down modulation of CD4 expression on peripheral blood memory CD4 T cells. C and D, MTRX1011A administration resulted in a dose dependent occupancy of CD4 sites on peripheral blood memory CD4 T cells.

Immediately following dose administration, the expression of CD4 on peripheral blood T cells was dose dependently decreased. Maximum down modulation of CD4 was approximately 75% and observed in the cohorts receiving 1, 3.5 and 7 mg/kg MTRX1011A IV in the SAD phase. As plasma levels of MTRX1011A decreased over time, CD4 expression levels returned to pre-dose levels in a dose dependent manner. The remaining CD4 sites on peripheral blood T cells were occupied with MTRX1011A in a dose dependent manner. Twenty-four hours after MTRX1011A administration no available CD4 sites on peripheral blood T cells were detected in the cohorts receiving 1, 3.5 and 7 mg/kg MTRX1011A IV, indicating complete CD4 receptor occupancy. MTRX1011A induced CD4 down-modulation and occupancy in the SC cohorts as well, but not to the same extent in the high dose IV groups. In contrast to prior studies with the depleting anti-CD4 antibody cM-T412, in which preferential PD effects were observed on naïve T cells compared to memory T cells [22], in our study CD4 receptor down-modulation and CD4 receptor occupancy in MRX1011A treated groups was identical between naïve and memory CD4 T cell subsets. Figure 3 presents the effect on the PD effects of MTRX1011A on the memory CD4CD45RA- T cell subset. Weekly doses of 1.5 mg/kg MTRX1011A were not sufficient to maintain CD4 down-modulation and CD4 receptor occupancy during the dosing phase whereas in both of the 3.5 mg/kg SC and the 5 mg/kg IV weekly cohorts maximum PD effects of MTRX1011A were maintained between weekly dose administrations (Figure 3B, D).

#### Immunophenotyping

**Figure 4:**
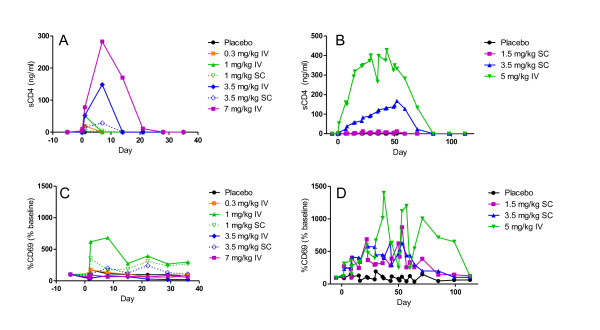
**Pharmacodynamic effects of MTRX1011A administration in peripheral blood**. Serum levels of soluble CD4-MTRX1011A complexes increased in a dose dependent fashion in the single dose phase (**A**) and multi-dose phase (**B**). MTRX1011A administration intravenously (IV) or subcutaneously (SC) lead to significant increase in CD69 expression on memory CD4 T cells in the single dose phase (**C**) and multi-dose phase (**D**).

To further characterize the effect of MTRX1011A on the immune system, we performed a phenotypical analysis of peripheral blood lymphocyte subsets. No effects were observed of MTRX1011A treatment on the number of memory or naïve T and B cell subsets. Strikingly, a significant increase in CD69 expression was observed on both naïve and memory CD4 T cell subsets during the dosing phase of MTRX1011A (Figure 4B). An additional activation marker, CD25 was not affected by MTRX1011A treatment on T cell subsets.

#### Serum analytes

CD4 is also present in the serum in a soluble form (sCD4) due to post-translational shedding from the surface of the cell [23]. Upon dose administration MTRX1011A bound to this soluble form of CD4, and elevated sCD4-levels were detected in the serum (Figure 4A). It was assumed that all sCD4 measured was in complex with MTRX1011A because of the molar excess of MTRX1011A to sCD4. Serum levels of CD4-MTRX1011A complexes dose-dependently increased in ascending dose cohorts and were approximately stable at steady state MTRX1011A exposure. Serum levels of the inflammatory cytokines GM-CSF, IFNγ, IL10, IL12p70, IL1β, LL2, IL6, IL8 and TNFα were not affected by MTRX1011A dosing, and levels of sCD25 were also not elevated (data not shown).

In summary, these PD biomarker data demonstrate that MTRX1011A dosing affects peripheral blood T cells in several ways. CD4 T cells were transiently reduced, CD4 receptor expression was down-modulated, sCD4 levels in serum were elevated and elevated CD69 expression was observed on CD4 T cells suggesting low levels of T cell activation without detection of concomitant cytokine release at the measured time points.

### Clinical activity

The effect of MTRX1011A on disease activity was evaluated only in the MD phase. A slightly higher proportion of patients in the 3.5 mg/kg SC MTRX1011A treated group achieved an ACR20, ACR50 and ACR70 response at eight weeks when compared to patients treated with placebo (Table 3). ACR20, ACR50 and ACR70 response rates were 33, 25, and 8%, respectively, for the 3.5 mg/kg MTRX1011A patients compared to 14, 0 and 0%, respectively, for the placebo treated patients. Patients receiving this dose demonstrated improvements in the mean SJC, which were reduced by 12 (60%) at Week 8 compared to baseline, and in mean TJC, which were reduced by 23 (56%) at Week 8 compared to baseline. Additionally, MTRX1011A treatment led to some minor improvement in DAS-CRP, DAS-ESR, ESR, and pain intensity; however, these improvements were also observed in patients treated with placebo and were not considered clinically significant. Taken together these assessments of disease activity suggest that treatment with 3.5 mg/kg MTRX1011A weekly for eight doses may lead to modest improvement in some markers of disease activity.

**Table 3:** Clinical activity assessments in the multi dose phase at eight weeks

	Placebo		MTRX1011A 1.5 mg/kg SC		MTRX1011A 3.5 mg/kg SC		MTRX1011A 5.0 mg/kg IV	
	baseline	eight weeks	baseline	eight weeks	baseline	eight weeks	baseline	eight weeks
ACR20, % patients		14		8		33		25
ACR50, % patients		0		0		25		0
ACR70, % patients		0		0		8		0
DAS-CRP	5.4 (4.3 to 6.6)	4.4 (3.2 to 5.9)	5.7 (4.1 to 6.7)	5.6 (4.0 to 7.7)	5.9 (3.7 to 6.8)	4.6 (2.6 to 6.8)	6.5 (4.2 to 8.0)	5.5 (3.2 to 6.8)
CRP, mg/dL	1.7 (0.03 to 4.0)	1.1 (0.1 to 3.0)	0.8 (0.02 to 2.3)	1.1 (0.02 to 4.2)	1.1 (0.02 to 7.7)	1.7 (0.1 to 9.8)	2.1 (0.1 to 8.2)	1.1 (0.1 to 3.9)
ESR, mm/hour	47 (15 to 120)	36 (8 to 104)	32 (8 to 60)	30 (6 to 108)	39 (0 to 78)	34 (8 to 58)	61 (26 to 110)	51 (29 to 104)
SJC	7 (0 to 16)	7 (0 to 19)	14 (2 to 30)	18 (2 to 51)	20 (7 to 52)	8 (0 to 36)	26 (3 to 59)	17 (0 to 30)
TJC	36 (10 to 53)	25 (3 to 62)	42 (12 to 65)	37 (4 to 67)	41 (8 to 68)	18 (0 to 66)	48 (22 to 65)	30 (24 to 33)
VAS,								
Patient, pain	60 (42 to 77)	39 (19 to 57)	57 (17 to 79)	59 (10 to 89)	62 (18 to 91)	54 (14 to 89)	78 (62 to 90)	47 (20 to 67)
Patient, overall	69 (54 to 94)	43 (18 to 75)	63 (17 to 98)	58 (17 to 88)	70 (40 to 93)	56 (10 to 87)	81 (60 to 100)	52 (25 to 75)
VAS, Physician, global	60 (26 to 78)	44 (25 to 63)	51 (21 to 80)	52 (12 to 88)	66 (35 to 100)	51 (13 to 88)	58 (45 to 76)	52 (33 to 73)

Data are expressed as group mean, with ranges depicted between parentheses. ACR, American College of Rheumatology; CRP, C-reactive protein; DAS, disease activity score; ESR, erythrocyte sedimentation rate; IV, intravenous; SC, subcutaneous; SJC, swollen joint count; TJC, tender joint count; VAS, visual analog scale.

## Discussion

In this study, we report the safety, tolerability, pharmacokinetics, and pharmacodynamics of MTRX1011A in patients with RA. MTRX1011A was tolerated up to doses of 1.5 mg/kg administered weekly SC for eight doses; however, at doses of 3.5 mg/kg SC weekly and 5.0 mg/kg IV weekly a moderate erythematosus papular pruritic rash was observed in some patients and considered dose limiting for this clinical indication. Though apparent maximum CD4 down-modulation and full CD4 occupancy were achieved and maintained in the 3.5 mg/kg SC MD cohort, only modest clinical activity was observed.

Overall, the results from this study are in agreement with previously reported studies evaluating safety, tolerability and efficacy of anti-CD4 treatments. Some evidence of clinical activity has been demonstrated with earlier anti-CD4 antibodies 4162W94, and keliximab, whereas a randomized Phase I/II study with clenoliximab demonstrated significant improvement in ARC20 responses [15-17]. Despite achieving maximum PD activity in peripheral blood in the weekly 3.5 mg/kg SC and 5 mg/kg IV dose groups, the clinical benefit of MTRX1011A treatment was only modest in the current study. Moreover, in three patients in the MD study treated with MTRX1011A a subjective worsening in disease activity was reported. Interestingly, in these patients CD4 down-modulation and occupancy was not maintained between dose administrations (data not shown). It is possible that studies investigating longer treatment duration could have shown more pronounced clinical efficacy had these dose levels been well tolerated.

The main objective of this Phase I study was to assess safety and tolerability, and 1.5 mg/kg SC weekly of MTRX1011A was established as the maximum tolerated dose. Given the known relationship between PD activity and efficacy, CD4 expression and occupancy PD assessments were incorporated to evaluate if MTRX1011A was able to achieve these predefined PD criteria at safe and tolerated dose levels. In this small phase I study, we were able to clearly demonstrate that the maximum tolerated dose of 1.5 mg/kg SC weekly was not sufficient to maintain the required maximum PD between dose administrations, and therefore, further development of MTRX0111 for the treatment of patients with RA was halted.

Incorporating measurements of PD activity in early clinical studies can support decision making and ensure that only molecules with increased chances of success will progress to the next stage of development. A thorough understanding of the mechanism of action of a therapeutic based on preclinical studies is critical to start with, and translating this knowledge to early clinical development and exploring detailed pharmacological measurements in human disease will ultimately benefit patients by providing safe and effective novel therapeutics.

Most previous anti-CD4 therapeutics have been associated with increased incidence of rash, and in this study MTRX1011A also induced a dose-limiting moderate pruritic rash [15,17,20]. The occurrence of rash is evidently associated with direct targeting of the CD4 molecule, but the mechanism of rash formation is not understood. The observation that the rash occurred typically after the third or fourth dose administration and was not limited to the injection site is indicative that an immunological response plays a role in its formation. We incorporated extensive pharmacological measurements to try to elucidate the mechanism by which MTRX1011A could cause rash in some patients. The observed increase in CD69 expression on T cells clearly demonstrates that *in vivo *MTRX1011A administration leads to an immediate and dose dependent early activation of T cells. In patients experiencing rash, the mean CD69 expression was slightly higher than that of other patients; however, given the small number of incidences observed in the study, we were not able to demonstrate a clear correlation between the extent of CD69 up-regulation and rash formation. Neither did we observe a clear correlation between the increased sCD4 levels following MTRX1011A administration and rash formation. Additional potential correlations between cases of rash and baseline CD4 counts, anti-MTRX1011A antibodies, serum cytokine levels did not identify a causal relationship (data not shown). The biopsy result of pallisaded neutrophilic and granulomatous dermatitis (PNGD) in one patient represents an interesting finding that has been described as a rare entity with an unknown etiology associated with (1) multiple disorders--mostly RA as well as other connective tissue disorders, (2) exposure to medications, and (3) myeloproliferative disorder [24-27]. Deposition of immune complexes in dermal tissues and the presence of IgM and C3 in dermal vessels and at the dermal-epidermal junction in PNGD patients have been reported [26,27].

## Conclusions

This study demonstrated that MTRX1011A was tolerated at weekly SC doses up to 1.5 mg/kg for eight doses. However, at this dose, MTRX1011A did not maintain maximum PD responses between weekly administrations, and higher dose levels that maintained maximum PD response were associated with dose-limiting rash. The cause of the pruritic rash remains elusive but is clearly associated with targeting CD4. Furthermore, only modest reduction in disease activity was observed even at doses above the maximum tolerated dose when maximum PD was maintained. Although CD4 T cells are certainly involved in the pathogenesis of RA, data from this study and those published by others have shown that targeting CD4 T cells with an anti-CD4 antibody is associated with at best a modest improvement in clinical parameters. Furthermore, this study also provides a model for the use of pharmacodynamic biomarkers in early decision making in clinical development.

## Abbreviations

ACR: American College of Rheumatology; AE: adverse event; CCP: anti-cyclic citrullinated peptide; CL: mean clearance; CRP: C-reactive protein; DAS28-CRP: Disease Activity Score, C-reactive protein; DAS-ESR: Disease Activity Score, erythrocyte sedimentation rate; DMARD: disease-modifying antirheumatic drug; ESR: erythrocyte sedimentation rate; FcRn: neonatal Fc receptor; IV: intravenous; K_D_: dissociation constant; mAb: monoclonal antibody; MD: multiple dose; MHCII: major histocompatibility complex class II; MTX: methotrexate; NSAIDs: nonsteroidal anti-inflammatory drugs; PD: pharmacodynamic; PK: pharmacokinetic; PNGD: pallisaded neutrophilic and granulomatous dermatitis; RA: rheumatoid arthritis; RF: rheumatoid factor; SAD: single ascending dose; SAE: serious adverse event; SC: subcutaneous; sCD4: CD4 soluble form; SJC: swollen joint count; TJC: tender joint count; VAS: visual analog scale

## Competing interests

The following authors are all employed by Genentech: HS, ZS, BI, MT, YZ, ES and JD. VC has not received any financial support and has no financial interest or conflict of interest with regard to the work described in our manuscript. CB has been a consultant for Genentech, Roche, and has received Grant Support from Genentech, Roche

## Authors' contributions

HS drafted the manuscript, generated PK/PD data and performed data analysis and trial design. ZS performed trial design, data analysis and drafted the manuscript. BI and CB performed the trial design and reviewed the manuscript. MT and ES performed trial design, generated PK/PD data and reviewed the manuscript. YZ generated PK/PD data and reviewed the manuscript. VC was the principal investigator and reviewed the manuscript. JD performed trial design, was the medical monitor, drafted the manuscript, reviewed the manuscript and performed data analysis. All authors have reviewed and approved the final manuscript for publication.
